# Are liver and renal lesions in East Greenland polar bears (*Ursus maritimus*) associated with high mercury levels?

**DOI:** 10.1186/1476-069X-6-11

**Published:** 2007-04-17

**Authors:** Christian Sonne, Rune Dietz, Pall S Leifsson, Gert Asmund, Erik W Born, Maja Kirkegaard

**Affiliations:** 1 Section for Contaminants, Effects and Marine Mammals, Department of Arctic Environment, National Environmental Research Institute, University of Aarhus, Frederiksborgvej 399, POBox 358, DK-4000 Roskilde, Denmark; 2 Department of Veterinary Pathobiology, Faculty of Life Sciences, University of Copenhagen, Bülowsvej 17, DK-1870 Frederiksberg, Denmark; 3 Greenland Institute of Natural Resources, P.O. Box 570, DK-3900 Nuuk, Greenland

## Abstract

**Background:**

In the Arctic, polar bears (*Ursus maritimus*) bio-accumulate mercury as they prey on polluted ringed seals (*Phoca hispida*) and bearded seals (*Erignathus barbatus*). Studies have shown that polar bears from East Greenland are among the most mercury polluted species in the Arctic. It is unknown whether these levels are toxic to liver and kidney tissue.

**Methods:**

We investigated the histopathological impact from anthropogenic long-range transported mercury on East Greenland polar bear liver (n = 59) and kidney (n = 57) tissues.

**Results:**

Liver mercury levels ranged from 1.1–35.6 μg/g wet weight and renal levels ranged from 1–50 μg/g wet weight, of which 2 liver values and 9 kidney values were above known toxic threshold level of 30 μg/g wet weight in terrestrial mammals. Evaluated from age-correcting ANCOVA analyses, liver mercury levels were significantly higher in individuals with visible Ito cells (p < 0.02) and a similar trend was found for lipid granulomas (p = 0.07). Liver mercury levels were significantly lower in individuals with portal bile duct proliferation/fibrosis (p = 0.007) and a similar trend was found for proximal convoluted tubular hyalinisation in renal tissue (p = 0.07).

**Conclusion**

Based on these relationships and the nature of the chronic inflammation we conclude that the lesions were likely a result of recurrent infections and ageing but that long-term exposure to mercury could not be excluded as a co-factor. The information is important as it is likely that tropospheric mercury depletion events will continue to increase the concentrations of this toxic heavy metal in the Sub Arctic and Arctic marine food webs.

## Background

Approximately 200–300 tons mercury (Hg) is yearly transported to the Arctic from lower latitudes via the atmosphere and large scale ocean currents [1,2], hence the Arctic acts as a sink for global emitted mercury due to spring mercury depletion events [3-6]. Mercury is toxic and the organic methyl-mercury (MeHg) form mediates central nervous system toxicity pre- and postnatally mainly via the posterior cortex [7,8]. Mercury levels are high in the Greenland and Faroe populations and a special focus has been to address health effects in these regions as such effects are proposed to have a significant socioeconomic impact [9].

The inorganic form of mercury is toxic to liver and kidney tissues due to the co-enzyme inhibition via high affinity to various microsome and mitochondria SH-group enzymes [8]. Studies of Atlantic bottlenose dolphins (*Tursiops truncatus*) [10] and Arctic beluga whales (*Delphinapterus leucas*) [11] have linked mercury exposure to liver histopathological changes. Also, renal glomerular and tubular lesions have been associated with mercury toxicity in controlled laboratory studies [12-14], in humans [15,16], in wildlife [11,17] and in domestic mammals [18,19].

In the Arctic, polar bears (*Ursus maritimus*) bio-accumulate mercury as they prey on polluted ringed seals (*Phoca hispida*) and bearded seals (*Erignathus barbatus*) which results in a high mercury uptake [1,2,20]. Studies have shown that East Greenland polar bears are among the most mercury polluted species in the Arctic [1,2]. In target organs like liver and kidney, inorganic mercury concentrations of 2.13–13.4 and 2.87–32.0 μg/g w.w. (wet weight) have been reported [21,22]. These levels exceed threshold levels for lethal mercury toxicity [1,2]. Although mercury is a naturally occurring element in the Arctic, Dietz et al. [23] showed that up to 94% of the mercury in East Greenland polar bears is of anthropogenic origin. We therefore examined liver and kidney histology and mercury concentrations in East Greenland polars sampled during 1999 to 2002 to determine whether high mercury concentrations may cause pathological changes at the histological level in target organs of large Arctic top predators.

## Methods

### Sampling

Liver (n = 59) and kidney (n = 57) samples were taken by local subsistence hunters in the Scoresby Sound area in central East Greenland (69°00'N to 74°00'N) during 1999–2002. A randomly chosen single renal lobe and a tissue sample from the periphery of a right liver lobe was taken for histological examination and fixed in a phosphate buffered formaldehyde/alcohol solution (3.5% formaldehyde, 86% ETOH and 10.5% H_2_O) to avoid freeze damage. In addition, sub samples for mercury analyses were stored in separate Polyethylene plastic bags until arrival at the laboratory in Roskilde. All samples were taken <12 *h post mortem*.

### The sample

The sample consisted of 32 subadults (10 females and 22 males), 15 adult females (5 ≥ 15 years) and 12 adult males (4 ≥ 15 years). Of these, 32 were caught during summer (1 June to 30 September) and 27 during winter (1 October to 31 May). The general East Greenland polar bear liver and renal histology, including micrographs, is described by Sonne et al. [24,25].

### Age estimation

The age determination was carried out by counting the cementum Growth Layer Groups (GLGs) of the lower I_3 _tooth after decalcification, thin sectioning (14 μm) and staining (toluidine blue) using the method described by Dietz et al. [26]. Adult males were categorised as ≥ 6 years, adult females as ≥ 5 years, old as ≥ 15 years and the remaining as subadults [27,28]. Furthermore, seasonal differences were investigated as summer: 1 June to 30 September vs. winter: 1 October to 31 May [27].

### Liver histology

The liver tissue was trimmed, processed conventionally, embedded in paraffin, sectioned at about 4 μm and stained with Haematoxylin (Al-Haematein)-Eosin (HE) and periodic acid-Schiff (PAS) for routine diagnostics, Van Gieson and Masson Trichrome to detect fibrous tissue (collagen), Best's carmine to demonstrate glycogen storage, Sudan III to detect lipid (frozen tissue) and Perls' Prussian blue reaction and Smorl for detecting haemosiderin and lipofuscin pigments, respectively [29,30].

Six histological features were evaluated: 1. Portal mononuclear cell infiltrations (absent, unifocally, multifocally or diffuse). 2. Random mononuclear cell infiltrations (average no. in 5 fields at 10× magnification). 3. Lipid granulomas (average no. in 5 fields at 10× magnification). 4. Hepatocytic steatosis (intracellular lipid; absent, foamy, multifocally macrovesiculary or diffuse macrovesiculary). 5. Visible Ito cells (average no. in 5 fields at 20× magnification). 6. Mild multifocally bile duct proliferation accompanied by portal fibrosis (absent or present). Each histological change was grouped semi-quantitatively as: 1. Portal mononuclear cell infiltrations: absent = 0; mild = unifocally; moderate = multifocally and severe = diffuse. 2. Random cell infiltrations: absent = 0; mild = Index ]0;1]; moderate = Index ]1;3] and severe = Index ]3;8]. 3. Lipid granulomas: absent = 0; mild = Index ]0;1 [; moderate = Index [1;2[and severe = Index [2;5]. 4. Hepatocytic steatosis: mild = foamy; moderate = multifocally macrovesiculary and severe = diffuse macrovesiculary. 5. Ito cells: absent = 0; mild = Index ]0;10]; moderate = Index ]10;50] and severe = Index ]50;200]. 6. Bile duct proliferation; see above.

### Renal histology

Kidney tissue was trimmed, processed conventionally, embedded in paraffin, sectioned at about 4 μm and Periodic acid-Schiff (PAS) and periodic acid silver methenamine (PAS-M) were used to demonstrate glomerular (capillary and mesangial) and proximal convoluted tubular changes; Van Gieson and Masson Trichrome to detect fibrous tissue (collagen) in the glomeruli (glomerulofibrosis) and in the interstitium (interstitial fibrosis) [30]. Seven histological features were evaluated: 1. Glomerular capillary wall thickening (10 randomly selected 5–40× fields). 2. Glomerular mesangial deposits (10 randomly selected 5–40× fields). 3. Tubular epithelial cell hyperplasia (10 randomly selected 5–40× fields). 4. Proximal convoluted tubular hyalinization/atrophy/dilations/necrosis (10 randomly selected 5–40× fields). 5. Tubular medullar hyaline casts (10 randomly selected 5–40× fields). 6. Interstitial fibrosis (10 randomly selected 5–40× fields). 7. Mononuclear cell infiltrations (10 randomly selected 5–40× fields). Each histological change was grouped semi-quantitatively as: 1. Glomerular capillary wall thickening: absent, mild and moderate. 2. Glomerular mesangial deposits: absent, mild and moderate. 3. Tubular epithelial cell hyperplasia: mild: focally; moderate: multi focally. 4. Proximal convoluted tubular hyalinization/atrophy/dilations/necrosis: mild: focally; moderate: multi focally. 5. Tubular medullar hyaline casts: mild: focally; moderate: multi focally. 6. Interstitial fibrosis: mild: focally; moderate: multi focally. 7. Mononuclear cell infiltrations: mild: focally; moderate: multi focally.

### Analyses of mercury

Liver (*n *= 59) and kidney (*n *= 57) samples were analysed for mercury levels (μg/g w.w.) according to Dietz et al. [23]. The principle was atomic absorption spectrometry (AAS; hydride generation and the flow injection analyses) have previously been described by Asmund et al. [31]. The detection limit was 0.005 mg/kg of dry weight. Analytical quality was ensured by repeated analyses and by frequent analysis of various certified reference materials [TORT-2 (lobster hepatopancreas), DORM-2, and Dolt-3] supplied by the National Research Council of Canada (Marine Analytical Chemistry Standards Program).

### Statistical analyses

The statistical analyses were performed with the SAS statistical software package (SAS V8 and enterprise guide V1) and the level of significance was set to *p *≤ 0.05, while significance at 0.05 <*p *≤ 0.10 was considered a trend. The mercury data were log-transformed (base e) prior to the analyses in order to meet the assumption of normality and homogeneity of the variance.

For each specific histologic change, a One-way ANOVA was performed to test for differences in mean age between individuals with and without that specific histologic change (Table 1). In case of hepatocytic steatosis, foamy cytoplasm was tested against macro vesicular lipid accumulation. Furthermore, we tested whether there was a relationship between sex and season ([summer: 1 June to 30 September]; [winter: 1 October to 31 May]), respectively, and histologic lesions using a χ^2^-test. In case of age dependency, a χ^2^-test was performed within subadults, adults and old, respectively, to test for sex differences.

**Table 1:** Prevalence (% [n]) of liver and kidney histopathology in East Greenland polar bears sampled during 1999–2002

**Organ**	**Absent**	**Mild**	**Moderate**	**Severe**	**Age *p *(F)**
*Liver (n = 59)*					
Multifocally mononuclear cell infiltrations	89 (53)	11 (6)	-	-	n.s.
Portal mononuclear cell infiltrations	85 (50)	8 (5)	7 (4)	-	n.s.
Lipid granulomas	37 (22)	36 (21)	22 (13)	5 (3)	n.s.
Hepatocytic steatosis	-	24 (14)	24 (14)	52 (31)	n.s.
Ito cell lipid accumulation	36 (21)	14 (8)	19 (11)	31 (19)	** (7.8)
Bile duct proliferation with fibrosis	92 (54)	8 (5)	-	-	*** (15.6)
*Kidney (n = 57)*					
Glomerular diffuse capillary wall thickening	72 (41)	25 (14)	3 (2)	-	*** (8)
Glomerular mesangial deposits/sclerosis	22 (13)	39 (22)	39 (22)	-	* (5.6)
Interstitial fibrosis	75 (42)	12 (7)	13 (8)	-	*** (49.8)
Tubular epithelial cell hyperplasia	75 (42)	12 (7)	13 (8)	-	** (6.7)
Tubular hyalinization/atrophy/dilatation/necrosis	61 (35)	12 (7)	27 (15)	-	*** (30.8)
Tubular medullar hyaline casts	84 (48)	13 (8)	3 (1)	-	* (6.6)
Mononuclear cell infiltrations	39 (22)	42 (24)	19 (11)	-	n.s.

Lesions are divided into groups of absent, mild, moderate and severe. n.s.: individuals with lesions not significantly older (mean age) than individuals without lesions at p > 0.05. *: individuals with lesions significantly older (mean age) than individuals without lesions at p ≤ 0.05, **: at p ≤ 0.01 and ***: at p ≤ 0.001.

The difference between mercury concentration and sex was tested in an age-normalizing analysis of covariance (ANCOVA) with mercury concentration as the dependent variable, age as covariable and sex as class variable, including their 1^st ^order interaction links (age × sex). After a successive reduction of non-significant interactions, judged from the type-III sum of squares (*p *≤ 0.05), the significance of each of the remaining factors was evaluated from the final model Least Square Mean (LSMean). Then, a One-way ANOVA was performed to test for differences in mercury mean concentration between subadults, adult females and adult males. The results were finally evaluated from Tukey's *post hoc *test (Table 2). In order to test the relationship between concentrations of mercury and age, a linear regression model was employed for subadults, adult females and adult males, respectively (Table 2).

**Table 2:** Mercury concentrations [mean ± SD (n), μg/g w.w.] in East Greenland polar bears

**Organ**	**Subadults**	**Adult females**	**Adult males**
Liver	6.27 ± 2.83 (32)*#	15.78 ± 8.6 (15)*	14.99 ± 8.96 (12)*
Kidney	7.18 ± 3.32 (31)*##	18.04 ± 10.97 (15)*	29.42 ± 14.09 (11)*,**

*: Significantly higher when compared to subadults at *p *≤ 0.05. **: Significantly higher when compared to adult females at p < 0.05. *#: significantly positive relationship to age at *p *≤ 0.002 (R^2 ^= 0.27). *##: significantly positive relationship to age at *p *≤ 0.001 (R^2 ^= 0.45).

Finally, the relationship between mercury concentration and each histologic change (absent vs. present) was tested by an age correcting analysis of covariance (Table 3). This was conducted with mercury concentration as dependent variable, age as covariable and histologic change (absent vs. present) as class variable, including their 1st order interaction links (age × histologic change). The statistical analyses were employed on all pooled individuals and additionally on subadults and adults, respectively. After a successive reduction of non-significant interactions, judged from the type-III sum of squares (*p *≤ 0.05), the significance of each of the remaining factors was evaluated from the final model Least Square Mean (LSMean).

**Table 3:** Significant results when testing histopathology vs. mercury levels in East Greenland polar bears

**Age Group**	**Organ**	**Histopathology**	***p (n, F, R*^2^*)***
All	Liver	Ito cell*	0.01 (50; 6.4; 0.53)
Adults	Liver	Lipid granulomas*	0.07 (22; 3.6; 0.38)
All	Liver	Portal bile duct proliferation and fibrosis**	0.007 (50; 8; 0.47)
Subadults	Kidney	Tubular hyalinisation, atrophy, dilatation and necrosis**	0.07 (31; 3.4; 0.51)

The calculations of differences between individuals with and without lesions are based on ANCOVA Least Square Mean regression analyses normalising for age. *: individuals with lesions higher in mercury levels than individuals without lesions. **: individuals with lesions lower in mercury levels than individuals without lesions.

## Results

### Liver lesions

Multifocal mononuclear cell infiltrations (lymphocytes, and macrophages and neutrophils) were found in 11%, portal mononuclear cell infiltrations in 15% and lipid granulomas in 63% of the bears, respectively (Table 1). All animals showed hepatocytic foamy cytoplasm (microvesicular steatosis), while 64% presented lipid accumulating Ito cells and 76% exhibited macrovesiculary steatosis (lipid vacuoles) in the periacinar zone 2 and 3. Mild bile duct proliferation accompanied by portal fibrosis was found in 8% of the animals. Only Ito cell lipid accumulation and bile duct proliferation were associated with age (both: p < 0.01) while none of the lesions were related to sex or season (all: p > 0.05).

### Renal lesions

Glomerular diffuse capillary wall thickening (similar to membranous glomerulonephritis due to immune deposits on the epithelial side of the glomerular capillary basement membrane) and PAS positive glomerular mesangial deposits/fibrosis was found in 28% and 78% of the animals, respectively (Table 1). Interstitial fibrosis, including dense Masson Trichrome and PAS-positive total fibrous obliteration of the glomerular sclerosis, was found in 25% of the individuals. All glomerular lesions exhibited positive age relationships (p < 0.05). Hyperplasia of distal convoluted tubule and collecting duct epithelial cells was found in 25% of the bears. PAS-positive hyalinization of the proximal convoluted tubular basement membrane accompanied by proximal convoluted tubular dilatation, atrophy, necrosis and interstitial fibrosis was found in 39% of the animals. In moderate cases, these lesions were accompanied by interstitial fibrosis and total glomerular obliteration. Tubular cylindrical hyaline casts (protein) were found in the medulla of 16% of the individuals, indicating protein loss. All tubular lesions exhibited positive age relationships (p < 0.05). The prevalence of mild and moderate interstitial fibrosis was 25%, while mononuclear cell infiltration in cortex, medulla and papilla was recorded in 61% of the polar bears. Mononuclear cell infiltrations were not related to age (p > 0.05). None of the renal lesions were related to sex or season (summer vs. winter) (all: p > 0.05).

### Mercury concentrations

**Figure 1:**
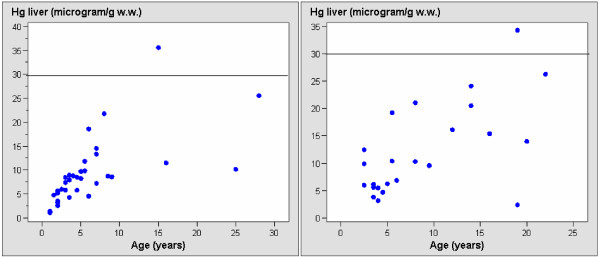
**Liver levels of mercury (μg/g w.w.) in 34 male (left) and 25 female (right) East Greenland polar bears**. Known threshold level for mercury toxicity in wildlife is given based on Dietz et al., (1998a) and AMAP (2005).

**Figure 2:**
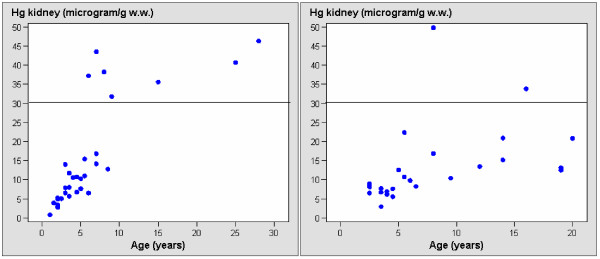
**Kidney levels of mercury (μg/g w.w.) in 32 male (left) and 25 female (right) East Greenland polar bears**. Known threshold level for mercury toxicity in wildlife is given based on Dietz et al., (1998a) and AMAP (2005).

Liver mercury levels ranged from 1.1–35.6 μg/g w.w. and renal levels ranged from 1–50 μg/g w.w., of which 2 liver values and 9 kidney values were above the known threshold toxic levels of 30 μg/g w.w. in terrestrial mammals (Table 2, Figure 1, 2). Mercury in subadults was significantly lower when compared to both males and females (p < 0.05), while kidney mercury levels in adult males were significantly higher when compared to adult females (p < 0.05). In general, the deviation was most pronounced in adult females. When analysing mercury vs. age relationship within subadults, adult females and adult males, respectively, the liver and kidney mercury increased significantly with age in subadults (both: p < 0.002), while that was not the case for adults. The analyses of covariance normalising for age revealed no difference in mercury concentrations when comparing all females and all males for both kidney (p > 0.05) and liver (p > 0.05) tissue, although it seemed that kidney concentrations decreased in old females and increased in old males. Neither was there a seasonal difference (summer vs. winter) in liver and kidney mercury concentrations (both: p > 0.05).

### Mercury concentrations and histological lesions

**Figure 3:**
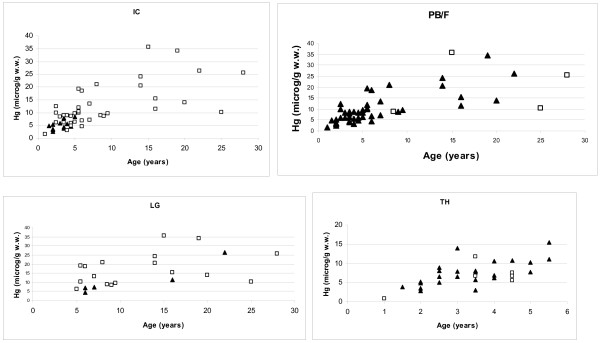
**Mercury concentration vs. age divided on histological lesions (yes: square; no: triangle)**. Visible Ito cells = IC; portal bile duct proliferation and fibrosis = PB/F; lipid granulomas = LG and proximal convoluted tubular hyalinisation = TH.

Based on these results, the relationship between histopathology (present vs. absent) and mercury was analysed on the entire pooled material and in subadults and adults, separately, within the age-correcting analyses of covariance. The analyses showed that mercury liver levels were significantly higher in individuals exhibiting visible Ito cells when compared to those not exhibiting visible Ito cells (p < 0.02) (Table 3, Figure 3). A similar trend was found for liver lipid granulomas (p > 0.05) (Table 3, Figure 3). In case of portal fibrosis, mercury levels were significantly lower in individuals exhibiting portal fibrosis when compared to those not exhibiting portal fibrosis (p = 0.007) and the same trend was found in case of hyalinisation of renal tubular basement membranes (p = 0.07) (Table 3, Figure 3).

## Discussion

### Liver histology

We found several different histopathological changes that could not be ascribed to specific etiological factors. The mononuclear cell infiltrates were non-specific inflammatory reactions towards micro organisms and/or injury of local blood vessels [32,33]. The bile duct proliferation and fibrosis were probably non-specific tissue reactions towards infections and thereby a result of ageing [32,33]. Prunescu et al. [34] speculated whether interstitial fibrosis was due to pre-hibernation physiological adaptations in brown bear (*U. arctos*). However, such a seasonal pattern could not be detected in the East Greenland polar bears. The number of lipid granulomas, in turn, was probably originated from Ito cell rupture. The lipid accumulation in polar bear Ito cells has previously been described and it constitutes the major accumulation and storage sites for lipophilic vitamin A [24,35-37]. Young bears only gradually start eating vitamin A rich prey until they are weaned at app. 2 years of age, which could explain the age difference in Ito cell numbers [36,37]. Specifically, the zonary (periacinary) hepatocytic steatosis could be ascribed to high lipid ingestion and starvation while we cannot rule out other co-factors such as abnormal hepatocytic function and decreased synthesis of apo proteins [32,33].

However, the considerable mercury concentrations accumulated in the liver tissue of the polar bears were in the range of adverse toxic effect levels for terrestrial mammals [1,2]. Specifically, hepatocytic steatosis was similar to mercury intoxication (hypoxia) in general [32,33]. The signs of chronic inflammation, bile duct proliferation, portal fibrosis and hepatocytic steatosis were similar to those reported in mercury exposed bottlenose dolphins [10] and Arctic beluga whales [11]. Studies on laboratory rodents support these findings [38]. Furthermore, Dietz et al. [23] showed that as much as 94% of the mercury in East Greenland polar bear hair sampled during 1892–2001 was likely of anthropogenic origin. Such an increase demands certain tolerance from polar bears to avoid sub cellular organ damage via *e.g*. up regulated methallothionein synthesis and selenid complex binding [1,2,10,40]. Previous studies of East Greenland polar bears have shown that the liver mercury:selenium molar ratio exceeded 1:1 for a few individuals which indicate that some of the total mercury is on a ion toxic form [22]. The fact that portal bile duct proliferation and fibrosis was associated with decreasing mercury concentrations may be due to decreased liver metabolism/function (uptake) or a simultaneously hepatocytic subcellular injury or death [39,41,42]. Furthermore, the positive association between mercury concentration and number of Ito cells and lipid granulomas could indicate a direct impact from mercury on the prevalence of liver inflammation [24]. In conclusion, the liver lesions were a result of recurrent infections and age while mercury could not be ruled out as potential co-factor.

### Renal histology

Diffuse thickening of the glomerular capillary wall, glomerular deposits/sclerosis and tubular changes are all well described in domestic animals [18,19], wildlife [11,17,40] and humans [15,16]. These lesions are all associated with age and recurrent infections while exposure to toxic substances is a known co-factor [15,16]. In the polar bears, glomerular sclerosis was worsen in cases of interstitial fibrosis, which is an expected and age-related change in various mammals, while the observed tubular epithelial cell hyperplasia has been associated with regeneration of renal parenchyma and chronic renal failure [15,16]. The interstitial nephritis (mononuclear cell infiltrations) was similar to those found in seals from North West Greenland and the Baltic [17,40] which we ascribe to chronic recurrent infections [15,16,18,19].

However, the histopathological lesions found in glomeruli (immune-complex glomerulonephritis), tubules and interstitium resemble those in Baltic grey seal and ringed seal heavily polluted with heavy metals between 1977–1996 [17]. Similar associations are also reported from controlled laboratory studies [12-16], in humans [15,16], in domestic mammals [18,19] and other Arctic wildlife species [11]. Proximal convoluted tubular basement membrane hyalinisation was associated with decreasing mercury levels. That could be due to a decreased metabolism/kidney function or because of mercury induced liver injury. Regarding the latter possibility, the quoted hepatic damage would increase the leakage of mercury (included in polypeptide complexes) into plasma and hence, it would also increase the burden of Hg filtered through the glomeruli, which in turn would generate tubular hyalinization [39,41,42] Proximal convoluted tubular basement membrane hyalinisation was associated with decreasing mercury levels. That could be due to a decreased metabolism/kidney function or because of mercury induced liver injury. Regarding the latter possibility, the quoted hepatic damage would increase the leakage of mercury (included in polypeptide complexes) into plasma and hence, it would also increase the burden of Hg filtered through the glomeruli, which in turn would generate tubular hyalinization [39,41,42]. According to Dietz et al. [22], the East Greenland polar bear renal tissue mercury:selenium molar ratio exceeded 1:1 for several individuals, which indicate that a large amount of the total mercury is on the ion toxic form, suggesting a toxic potential. However, the conclusion is that the chronic nature of the lesions was a result of age and recurrent infections while we cannot exclude mercury as a co-factor.

### Considerations

Whether the present lesions have an impact on health status at the individual level is impossible to evaluate. However, it cannot be excluded that individuals that are more susceptible to mercury toxicity may be affected as 16% and 3% of the animals exceeded renal and liver threshold levels, respectively, for toxic effects of the quoted metal in mammals [2]. Furthermore, Sonne et al. [24,25] showed that anthropogenic organochlorines and brominated flame retardants were possible co-factors in the development of renal and liver lesions in East Greenland polar bears. The present study shows associations between organ lesions and mercury levels which indicate mercury as a co-factor although recurrent infections and age clearly were main factors. Anyhow, the mercury levels adds another risk factor to polar bear health as the pollution cocktail increase and thereby the possibility of additive effects. Furthermore, the polar bear health mercury problem may be most pronounced in the Western Arctic, as the highest concentrations exists there [1,2,23] due to increasing emissions from Eurasia.

## Conclusion

We found liver and kidney mercury levels in East Greenland polar bears in the range of toxic effects. The signs and nature of chronic inflammation and statistical relationships points towards age and recurrent infections as main factor while mercury could not be ruled out as a possible co-factor. These are new and important results in the monitoring and assessment of the potential toxic impact from the increasing mercury concentrations in Arctic wildlife and humans relying on polluted marine species.

## Competing interests

The author(s) declare that they have no competing interests.

## Authors' contributions

CS drafted the manuscript. CS and RDI outlined the study design and RDI conducted the age determinations with MKI. PSL participated in the histopathological examinations. GA conducted the mercury analyses, EWB participated in the study design. All authors read and approved the final manuscript.
